# Seminal vesicle intrafraction motion analysed with cinematic magnetic resonance imaging

**DOI:** 10.1186/1748-717X-9-174

**Published:** 2014-08-08

**Authors:** Suki Gill, Kim Dang, Chris Fox, Mathias Bressel, Tomas Kron, Noelene Bergen, Nick Ferris, Rebecca Owen, Sarat Chander, Keen Hun Tai, Farshad Foroudi

**Affiliations:** Department of Radiation Oncology, Peter MacCallum Cancer Centre, Melbourne, Australia; Radiation Therapy Services, Peter MacCallum Cancer Centre, Locked Bag 1; A’ Beckett Street, Melbourne, Australia; Physical Sciences, Peter MacCallum Cancer Centre, Melbourne, Australia; Biostatistics and Clinical Trials, Peter MacCallum Cancer Centre, Melbourne, Australia; Department of Cancer Imaging, Peter MacCallum Cancer Centre, Melbourne, Australia; Department of Pathology, University of Melbourne, Melbourne, Australia

## Abstract

**Purpose:**

This study analyses seminal vesicle displacement relative to the prostate and in relation to treatment time.

**Method:**

A group of eleven patients undergoing prostate cancer radiotherapy were imaged with a continuous 3 T cine-MRI in the standard treatment setup position. Four images were recorded every 4 seconds for 15 minutes in the sagittal plane and every 6.5 seconds for 12 minutes in the coronal plane. The prostate gland and seminal vesicles were contoured on each MRI image. The coordinates of the centroid of the prostate and seminal vesicles on each image was analysed for displacement against time. Displacements between the 2.5 percentile and 97.5 percentile (i.e. the 2.5% trimmed range) for prostate and seminal vesicle centroid displacements were measured for 3, 5, 10 and 15 minutes time intervals in the anterior-posterior (AP), left-right (LR) and superior-inferior (SI) directions. Real time prostate and seminal vesicle displacement was compared for individual patients.

**Results:**

The 2.5% trimmed range for 3, 5, 10 and 15 minutes for the seminal vesicle centroids in the SI direction measured 4.7 mm; 5.8 mm; 6.5 mm and 7.2 mm respectively. In the AP direction, it was 4.0 mm, 4.5 mm, 6.5 mm, and 7.0 mm. In the LR direction for 3, 5 and 10 minutes; for the left seminal vesicle, it was 2.7 mm, 2.8 mm, 3.4 mm and for the right seminal vesicle, it was 3.4 mm, 3.3 mm, and 3.4 mm. The correlation between the real-time prostate and seminal vesicle displacement varied substantially between patients indicating that the relationship between prostate displacement and seminal vesicles displacement is patient specific with the majority of the patients not having a strong relationship.

**Conclusion:**

Our study shows that seminal vesicle motion increases with treatment time, and that the prostate and seminal vesicle centroids do not move in unison in real time, and that an additional margin is required for independent seminal vesicle motion if treatment localisation is to the prostate.

## Introduction

The seminal vesicles are included in the clinical target volume (CTV) for prostate cancer radiotherapy usually in patients deemed to have a risk of invasion above 15% according to Partin tables or the Roach equation [1, 2]. Although including the seminal vesicles in the CTV leads to a larger planning target volume (PTV) and irradiation of a larger volume of adjacent rectum and bladder, with modern techniques such as intensity-modulated radiotherapy (IMRT), including the seminal vesicles has been shown to be achievable with only a small increase in normal tissue complication probability [3]. However, IMRT, volumetric modulated arc therapy and stereotactic body radiotherapy have sharp dose gradients outside the intended target, and an accurate assessment of radiotherapy margins is necessary.

Image-guided radiotherapy (IGRT) in prostate cancer improves the accuracy of treatment delivery and has been shown to reduce toxicity [4, 5]. IGRT for prostate cancer can be conducted with pretreatment imaging and registration to fiducial markers implanted in the prostate which corrects for interfraction displacement [4, 6]. Interfraction motion of the seminal vesicles has been reported previously by assessing serial pretreatment images taken during prostate cancer radiotherapy [7–9]. However with IGRT localization to the prostate, it is uncertain if this same localization applies to the seminal vesicles and therefore the same CTV to PTV margins can be used for intrafraction motion of the seminal vesicles.

Cine MRI is a useful modality which can continually monitor motion of internal organs without exposing the patient to ionizing radiation. It has been used previously to study the intrafraction motion of the prostate [10–15]. We conducted the present study to analyse if the magnitude of displacement of the seminal vesicles was similar to that of the prostate, to see if the same margins could be applied in the setting of IGRT.

## Method

This study was approved by the local institutional ethics board Peter MacCallum Cancer Centre Ethics Committee prior to commencement. Prior to starting radiotherapy for prostate cancer, eleven patients underwent a cine-MRI in a time frame compatible with a standard IGRT fraction delivery time. Disease stage for each patient are shown in Table 1. All patients were instructed to empty their bladder and bowel one hour before the scan and then to drink 750 mls of water to follow the same department preparation protocol as prior to a standard prostate radiotherapy fraction. Patients were positioned as for radiotherapy delivery, supine on the MRI couch (flat table) and stabilized with a knee rest and foot stocks.

**Table 1:** **Disease stage for each patient**

Patient number	T stage	Gleason score	Pre-RT PSA
1	T3b	3 + 4	12.2
2	T2c	3 + 4	19.1
3	T2c	3 + 4	20.6
4	T2b	4 + 4	15.9
5	T1c	3 + 4	12.4
6	T1c	3 + 3	12.5
7	T2b	4 + 3	18.3
8	T3b	4 + 3	9.3
9	T1c	3 + 3	7.6
10	T2a	3 + 3	7.5
11	T2b	3 + 4	7.8

**Figure 1:**
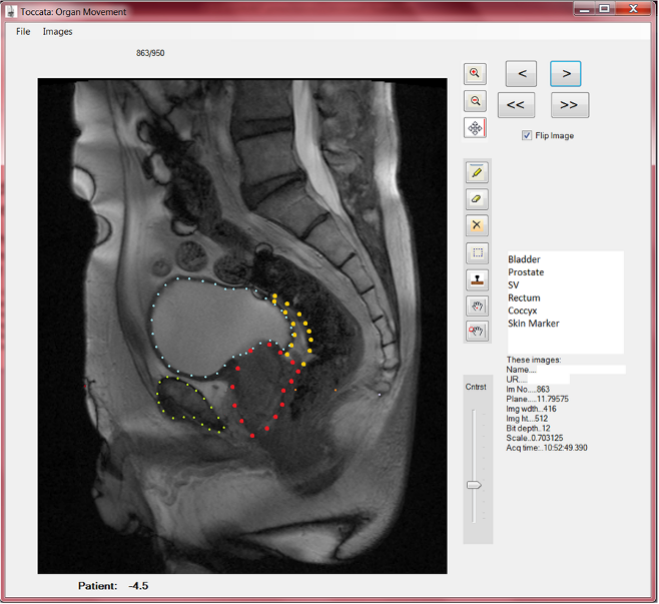
**Toccata; in house software on which the prostate and seminal vesicles were contoured, this example showing a sagittal image contour.**

All patients underwent a True-FISP (Fast Imaging in Steady-state Precession) T2 weighted cine-MRI scans on a Siemens 3-tesla Tim Trio MRI system (Siemens Medical Solutions, Malvern, PA). The MRI scanning protocol consisted of an alternation of three 3D volume and two cine-MRI acquisitions (Sagittal; TR-5.11 ms, TE-2.56 ms, Thick-5 mm, Space-10 mm, Slices-4, FOV-360 mm, Matrix-512×333, Voxel size-0.9×0.7×5.0 mm, Phase encoding direction-A > P, Bandwidth-543 Hz/Px and Coronal; TR-5.01 ms, TE-2.51 ms, Thick-5 mm, Space-10 mm, Slices-4, FOV-380 mm, Matrix-512×410 mm, Voxel size-0.9×0.7×5.0 mm, Phase encoding direction-R > L, Bandwidth-543 Hz/Px). With these parameters, the chemical shift is 440/543 pixels, or about 0.6 mm in the frequency encoding direction. We have previously published on our treatment times for prostate cancer IGRT [16]. The median time for IGRT treatment was 6 minutes for kilovoltage orthogonal imaging with automated couch shift versus 10 minutes for megavoltage orthogonal imaging and manual couch shift. Setup time ranged from 3.0 to 6.2 mins (mean 4.8 mins). Therefore prostate IGRT treatment time can vary from 9–16 minutes depending on equipment. Total MRI scan time was 30 minutes, of which 15 minutes were in the sagittal plane, and 12 minutes in the coronal plane. Four images were recorded every 4 seconds for 15 minutes in the sagittal plane followed by four images every 6.5 seconds for 12 minutes in the coronal plane. All coronal datasets represented a time frame of 12 minutes, because we were limited to a 30 minute appointment slot for each patient.Prostate and seminal vesicles were manually contoured in the sagittal and coronal planes on every frame using customised software written in VB.NET. The contouring was conducted by one investigator (KD) and checked by a second investigator (SG). This software catalogued the large number of images (up to 1000 per patient) and organised them according to the reconstructed plane. Tools were provided to draw and manipulate organ contours on these images and to save the contours to a text file. A second program analysed the contour information for each image from the text files to find either the centroid of a contour, the position of a point, or the distance across a contour along a specified axis (see Figure 1). The centroid position of the prostate and each seminal vesicle was analysed for each frame relative to the first frame. Anterior-posterior (AP) and superior-inferior (SI) displacement in time intervals (T) 3, 5, 10 and 15 minutes from the start were analysed on sagittal images. Left-right (LR) displacement in time intervals (T) 3, 5 and 10 minutes from the start were analysed on coronal images.

To study the effect of treatment time on prostate and seminal vesicle displacement, for each patient, the range from the 2.5 percentile to the 97.5 percentile (2.5% trimmed range) was calculated for the first 0 to 3 minutes, 0 to 5 minutes, 0 to 10 minutes and 0 to 15 minutes. The reason we used the 2.5% trimmed range instead of presenting the 95% confidence interval is because the confidence interval assumes an underlying probability density function (e.g. normal distribution) while our interest was to provide a summary measure to describe the magnitude of motion after removing the 5% most extreme values (2.5% of each side). We chose to use the 2.5% trimmed range instead of other popular trimmed ranges like the interquartile range (25% trimmed range) and the interdecile range (10% trimmed range) to exclude only the most extreme observation but still capture most of the data. We also believe the 2.5% trimmed range have better clinical interpretation (as it corresponds to the range for 95% of the displacement) than the interquartile range (range for 50% of the displacement).

The 2.5% trimmed ranges were compared between the four observation durations using Friedman’s test. The Wilcoxon test was used to compare the prostate and seminal vesicle range of displacement for each of the four treatment durations. The F-test was used to compare the variances of seminal vesicles and prostate range of displacement for each of the 4 treatment durations.

Spearman’s rank correlation was used to evaluate the relationship between prostate and seminal vesicles range of displacement. The Pearson product–moment correlation coefficient was used to evaluate the relationship between the movements of the prostate and seminal vesicles within patient, for each individual patient.

## Results

A total of 11 sagittal datasets and 10 coronal datasets were obtained, as one cine- MRI was terminated early by one patient. 10 out of 11 sagittal dataset were taken for a time length of 15 minutes and 1 dataset taken in 12 minutes.

### The effect of treatment duration on seminal vesicle displacement

On sagittal images, the average 2.5% trimmed range of displacements for 3, 5, 10 and 15 minutes for seminal vesicle centroids in the SI direction were 4.7, 5.8, 6.5, 7.2 mm respectively. In the AP direction, seminal vesicle the average 2.5% trimmed range of displacements was 4.0, 4.5, 6.5 and 7.0 mm respectively for 3, 5, 10 and 15 minutes. On coronal images, in the LR direction the average 2.5% trimmed range of displacements for the left seminal vesicle for the first 3, 5 and 10 minutes were 2.7, 2.8 and 3.4 mm and for the right seminal vesicle, it was 3.4, 3.3 and 3.4 mm respectively. The Friedman’s test showed that there was a difference in the 2.5% trimmed range of displacements for the seminal vesicles between the treatment’s durations for the sagittal plane (p = 0.001 for AP direction and P < 0.001 in SI direction). The average 2.5% trimmed range of displacements for the seminal vesicles significantly increases as the duration of the treatment increases for the SI direction using sagittal image for all treatment durations.

### The relationship between prostate and seminal vesicle displacement for the group

The means and standard deviations and results of the Wilcoxon test and F test comparing prostate and seminal vesicle 2.5% trimmed range of displacements are given in Table 2. The average range of prostate displacements and seminal vesicle displacements increases with treatment time, but this is more so for the seminal vesicles than the prostates. The seminal vesicles move, on average, more than the prostate in the sagittal plane for the SI direction but not for the AP direction and p values are as highlighted in Table 2. There was no difference in displacement between prostate, right seminal vesicle and left seminal vesicle for the LR direction.

**Table 2:** **Comparison of prostate and seminal vesicles range of displacements (2.5% trimmed range)**

Plane	Direction and duration	Prostate		SV		p-value (mean)	p-value (SD)
		Mean	SD	Mean	SD		
Saggital	AP 3 min	3.6	1	4	1.4	0.413	0.221
	AP 5 min	4.2	1.3	4.5	1.7	0.831	0.326
	AP 10 min	5.3	2.1	6.5	4.1	0.831	0.049
	AP 15 min	5.3	1.9	7	3.6	0.054	0.06
	SI 3 min	3.3	0.7	4.7	2.8	0.01	<0.001
	SI 5 min	4.4	1.8	5.8	3.4	0.042	0.083
	SI 10 min	5.1	1.5	6.5	3.9	0.067	0.009
	SI 15 min	5.3	1.6	7.2	4	0.019	0.009
Coronal	Lt SV LR 3 min	3	1.7	2.7	1.3	0.625	0.469
	Lt SV LR 5 min	3	1.4	2.8	1.4	0.695	0.92
	Lt SV LR 10 min	3.1	1.7	3.4	1.6	0.322	0.846
	Rt SV LR 3 min	3	1.7	3.4	1.9	0.557	0.694
	Rt SV LR 5 min	3	1.4	3.3	1.7	0.322	0.645
	Rt SV LR 10 min	3.1	1.7	3.4	1.8	0.322	0.864

The Wilcoxon test compares prostate and seminal vesicle displacement for each of the four treatment durations. The F-test was used to compare the variances of seminal vesicles and prostate displacement for each of the four treatment durations.

### The relationship between prostate and seminal vesicle motion for each patient

**Figure 2:**
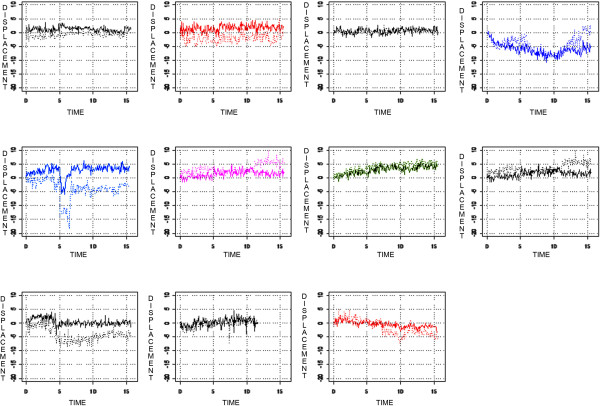
**Prostate AP displacement in millimeters (solid line) and seminal vesicle AP displacement (dotted line) in relation to its point of origin over time in minutes.** Each graph represents one patient.

**Figure 3:**
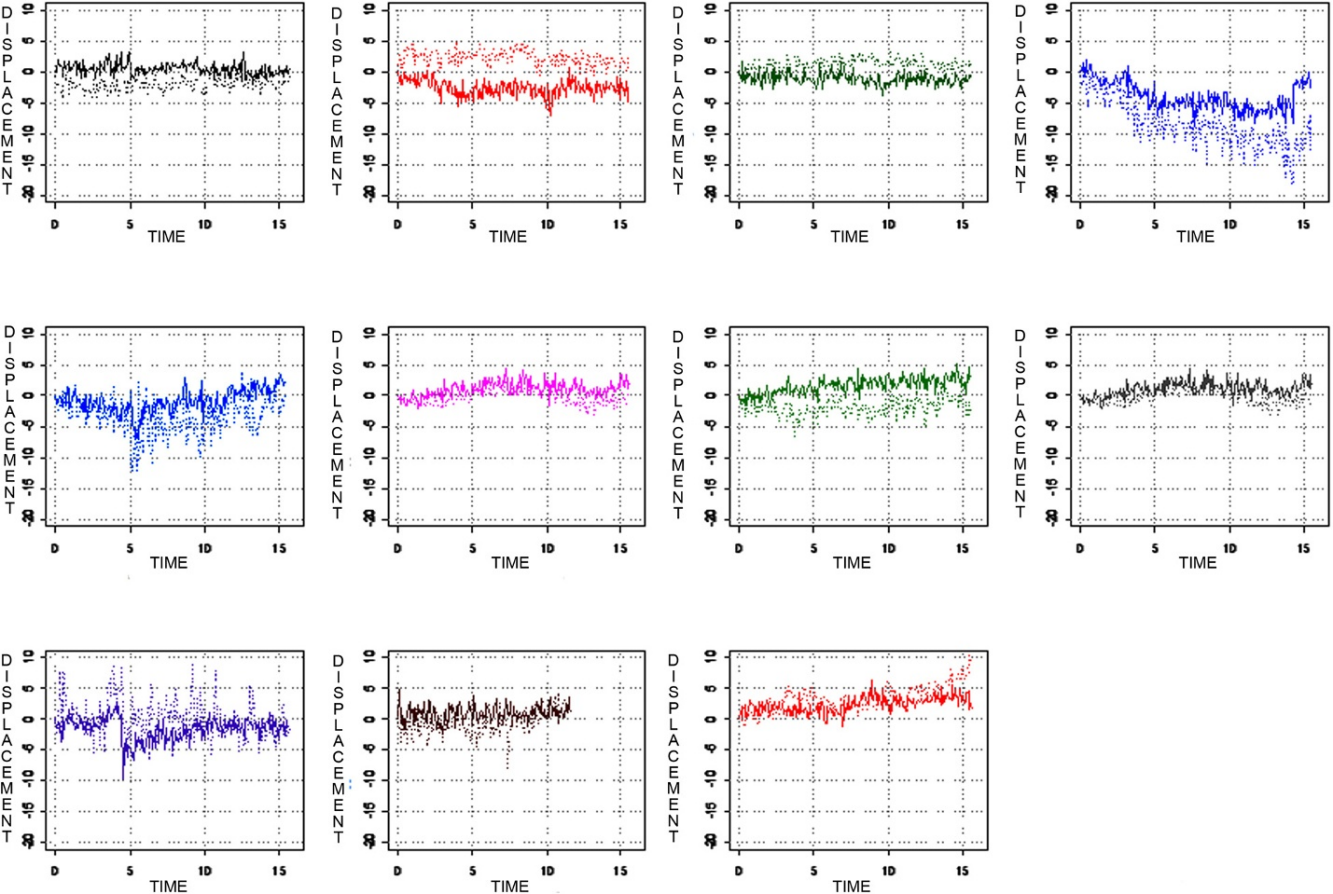
**Prostate SI displacement in millimeters (solid line) and seminal vesicle SI displacement (dotted line) in relation to its point of origin over time in minutes.** Each graph represents one patient.

**Figure 4:**
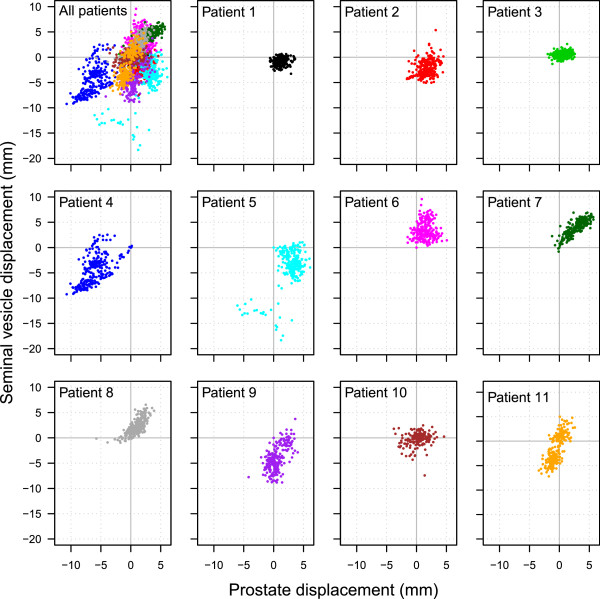
**The simultaneous displacement of the prostate and seminal vesicles at each timepoint compared to its point of origin in the AP direction.** Each graph represents one patient.

**Figure 5:**
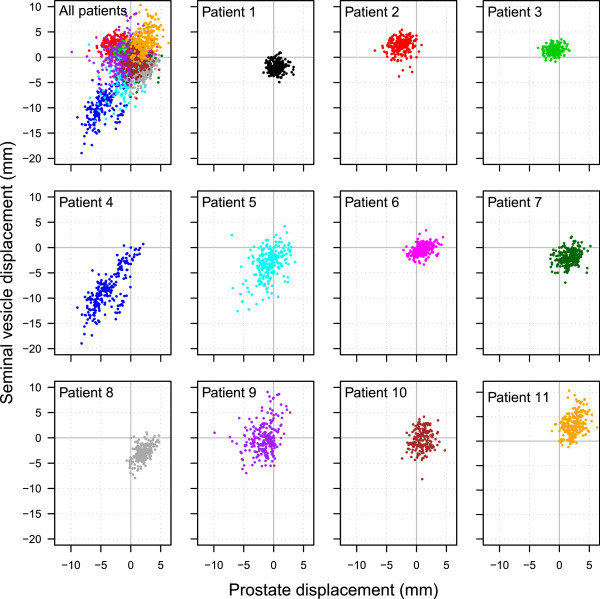
**The simultaneous displacement of the prostate and seminal vesicles at each timepoint compared to its point of origin in the SI direction.** Each graph represents one patient.

Figures 2 and 3 show the prostate and seminal vesicle displacement over time in the AP and SI directions for each patient. While some patients had a strong relationship between prostate and seminal vesicle displacement, others showed no relationship, indicating that the relationship was patient specific. Some patients had a displacement greater than 3 mm during a significant part of the treatment. Except for one patient (patient 4), displacement was hardly ever larger than 5 mm for the prostate in all directions (sagittal and coronal planes). Figures 4 and 5 show the displacement of the prostate and seminal vesicle at the same time-points compared to its point of origin in the AP and SI directions. The prostate and seminal vesicle displacement showed a linear trend for some patients, but for the majority there is a random spread, indicating that the displacement of the prostate was not related with the displacement of the seminal vesicles. Table 3 shows the Pearson product–moment correlation coefficient for real-time prostate and seminal vesicle displacement for each patient in the sagittal and coronal planes. The Pearson product–moment correlation coefficient varied substantially between patients indicating that the relationship between prostate displacement and seminal vesicles displacement is patient specific with the majority not having a strong relationship.

**Table 3:** **Pearson product–moment correlation coefficient between prostate and seminal vesicles displacement (within patients)**

	Sagittal		Coronal			
	Prostate vs. SV		Prostate vs. right SV		Prostate vs. left SV	
Patient	AP	SI	LR	SI	LR	SI
1	0.19	0.00	-	-	-	-
2	0.29	0.13	-0.28	0.28	-0.10	0.56
3	0.22	0.16	0.03	0.88	0.19	0.51
4	0.60	0.75	0.07	0.87	0.19	0.86
5	0.54	0.40	0.24	0.04	0.48	0.35
6	0.05	0.42	0.16	0.25	-0.23	0.07
7	0.82	0.23	0.09	0.35	0.13	0.35
8	0.72	0.57	0.11	0.72	0.23	0.22
9	0.61	0.29	0.29	0.28	0.15	0.29
10	0.17	0.12	0.04	0.01	-0.06	0.10
11	0.75	0.30	0.27	0.26	0.25	0.40

Note: The confidence interval varies depending on the correlation. The confidence interval for 0 correlation is [-0.13; 0.13], for 0.5 correlation is [0.40; 0.60] and for 0.8 is [0.75; 0.85].

## Discussion

Our analysis shows that the displacements in the SI direction was significantly more for seminal vesicles than for the prostate. Both the prostate and the seminal vesicle displacements increase with treatment time up to 10 minutes, but then appeared to plateau between ten and fifteen minutes. The observation that displacements plateaued after 10 minutes indicates that maximal displacements for these organs (i.e. the prostate and seminal vesicles) were reached, and that further displacements beyond the maximum displacement is less likely to be seen after ten minutes. This has implications for radiotherapy margins; if treatment were given over time periods shorter than 10 minutes, for example with VMAT, then gains in terms of reduced margins are likely to be had. However close monitoring of prostate motion is more appropriate for longer treatment times, rather than larger margins. Motion in the LR direction was seen to be minimal, with average displacement around 3 mm. While some patients had a strong correlation between prostate and seminal vesicle displacement in the AP and SI direction during the course of the treatment, others showed no significant relationship, indicating that the relationship is patient specific. The prostate and seminal vesicle motion did not correlate in the LR direction.

From a clinical standpoint, our results have significant implications when considering radiotherapy margins for seminal vesicles especially in high risk prostate cancer where the seminal vesicles are included in the target volume. Studies looking at interfraction seminal vesicle motion show that the seminal vesicles move more than the prostate [9]. Interfraction seminal vesicle motion has been assessed by Frank et al. using serial pre-treatment CTs, who demonstrated that the mean 3D vector displacement for the prostate was 4.6 mm and for the seminal vesicle it was 7.6 mm [7]. Liang et al. studied seminal vesicle interfraction motion and found that minimum margins of 3 mm for prostate and 4.5 mm for SV were required for IMRT with prostate only image guidance [8]. Our study however is the first confirming that the intrafraction margin for seminal vesicles is also greater, which is the foremost uncertainty once interfraction motion is corrected for with IGRT of the prostate.

In addition, the movement of the seminal vesicles was weakly correlated with the movement of the prostate for most patients, which has implications for real time prostate tracking techniques. For example, the Calypso® transponder is being increasingly used for IGRT while tracking to the prostate, where the treatment can be stopped and patient repositioned if the prostate displaces above a predefined threshold. For example, using data from the Calyso® system, Curtis et al. estimated that 1, 2, and 3 mm vector planning margins require a respective imaging frequency of every 15, 60, and 240 seconds to account for intrafraction prostate motion while achieving adequate geometric target coverage for 95% of the time [17]. However if the seminal vesicles are also included in the volume, our study shows that additional margins are required for the seminal vesicles as tracking to the prostate is not a reliable surrogate for seminal vesicles. As radiotherapy techniques become more sophisticated, for example with better target delineation using MRI, better IGRT for example with MRI linacs, and even adaptive replanning to individualize treatment, there is an attempt to also re-define and reduce the CTV-PTV margins. However, the further understanding of the bio-mechanics of the target must guide the development of algorithms that are used in radiation planning and therapy to produce a meaningful PTV rather than just static geometric ones.

There are some of limitations of this study. Firstly CTV to PTV margins also include uncertainties other than geometrical displacement which have not been measured in this study. For example contouring uncertainty, mechanical limitations of the linear accelerator and software are also factored into the PTV margin [18]. Deformation of the seminal vesicles is also more prominent compared to deformation of the prostate [9], and because our software calculated displacement of the centroids of prostates and seminal vesicles intrafraction deformation is not measured in this study. In addition, although we have looked at 95% of displacements, the clinical relevance of a displacement becomes more significant if it is not momentary, and it is as yet undefined what duration of displacement is clinically significant. Future studies looking at seminal vesicle motion should also investigate dosimetric coverage.

## Conclusion

Seminal vesicles moved significantly more than the prostate in the SI direction, but not in the AP or LR direction. Prostate and seminal vesicle displacement increased with treatment time in the AP and SI direction. When setting up to the prostate, larger margins are required for seminal vesicles in the SI direction. The movement of the seminal vesicles were weakly correlated with the movement of the prostate, which has implications for real time prostate tracking techniques if the intent is to treat the seminal vesicles at the same time.

## Consent

All patients in this study provided written informed consent to the publication of this study.
